# Gut Microbiota Metabolism of Azathioprine: A New Hallmark for Personalized Drug-Targeted Therapy of Chronic Inflammatory Bowel Disease

**DOI:** 10.3389/fphar.2022.879170

**Published:** 2022-04-05

**Authors:** Slavica Lazarević, Maja Đanic, Hani Al-Salami, Armin Mooranian, Momir Mikov

**Affiliations:** ^1^ Department of Pharmacology, Toxicology and Clinical Pharmacology, Faculty of Medicine, University of Novi Sad, Novi Sad, Serbia; ^2^ The Biotechnology and Drug Development Research Laboratory, Curtin Medical School & Curtin Health Innovation Research Institute, Curtin University, Perth, WA, Australia; ^3^ Hearing Therapeutics Department, Ear Science Institute Australia, Queen Elizabeth II Medical Centre, Nedlands, WA, Australia

**Keywords:** drug metabolism, gut microbiome, thiopurine therapy, biotransformation, drug response, microbial metabolism, first-pass metabolism, precision medicine

## Abstract

Despite the growing number of new drugs approved for the treatment of inflammatory bowel disease (IBD), the long-term clinical use of thiopurine therapy and the well-known properties of conventional drugs including azathioprine have made their place in IBD therapy extremely valuable. Despite the fact that thiopurine S-methyltransferase (TPMT) polymorphism has been recognized as a major cause of the interindividual variability in the azathioprine response, recent evidence suggests that there might be some yet unknown causes which complicate dosing strategies causing either failure of therapy or toxicity. Increasing evidence suggests that gut microbiota, with its ability to release microbial enzymes, affects the pharmacokinetics of numerous drugs and subsequently drastically alters clinical effectiveness. Azathioprine, as an orally administered drug which has a complex metabolic pathway, is the prime illustrative candidate for such microbial metabolism of drugs. Comprehensive databases on microbial drug-metabolizing enzymes have not yet been generated. This study provides insights into the current evidence on microbiota-mediated metabolism of azathioprine and systematically accumulates findings of bacteria that possess enzymes required for the azathioprine biotransformation. Additionally, it proposes concepts for the identification of gut bacteria species responsible for the metabolism of azathioprine that could aid in the prediction of dose-response effects, complementing pharmacogenetic approaches already applied in the optimization of thiopurine therapy of IBD. It would be of great importance to elucidate to what extent microbiota-mediated metabolism of azathioprine contributes to the drug outcomes in IBD patients which could facilitate the clinical implementation of novel tools for personalized thiopurine treatment of IBD.

## Introduction

As the incidence of inflammatory bowel disease (IBD) is dramatically rising, both in the Western and developing countries, the arsenal of novel therapeutic options has also grown larger. The etiology of IBD still remains poorly understood with the main goal of the therapy being to induce and maintain remission, thus providing a better quality of life for patients. However, the novel class of biological drugs such as anti-TNF therapy and monoclonal antibodies fail to induce remission in approximately one-quarter of patients and also many have been associated with severe side-effects and drug interactions (Sandborn et al., 2019; Sanchis-Artero and Martínez-Blanch, 2020). Therefore, their cost-effectiveness has been critically taken under consideration even in developed countries (Pillai et al., 2017; de Boer, 2019).

On the contrary, reliance on the conventional therapy is present among clinicians due to extensive familiarity with its characteristics. 6—mercaptopurine (6-MP) and its prodrug azathioprine are thiopurine drugs that have been used widely for more than 50 years, thus they became the most commonly used immunomodulators. The European Crohn’s and Colitis Organization developed a consensus on the use of thiopurines as a monotherapy for the maintenance of remission or an adjunctive therapy to infliximab in Crohn’s disease (CD) and steroid-dependent Ulcerative colitis (UC), highlighting the importance of the thiopurines role in IBD therapy (Lim and Chua, 2018).

Nevertheless, a therapeutic response and toxicity of thiopurine therapy are highly variable among individuals and remain highly unpredictable (Citterio-Quentin et al., 2017). Pre-treatment testing of genetic polymorphism of enzyme thiopurine S-methyltransferase (TPMT) involved in the thiopurine metabolism is an important tool for optimizing thiopurine therapy. Moreover, therapeutic drug monitoring that includes the profiling of active and inactive drug metabolites, 6-thioguanine (6-TGN) and 6-methylmercaptopurine (6-MMP) offers an opportunity to optimize doses early. However, these approaches just partly explain the variability and unknown causes remain to be resolved (Duley and Florin, 2005; Moreau et al., 2014).

The growing list of known oral drugs susceptible to direct human gut bacterial metabolism has opened a new era of pharmacomicrobiomics (Lam et al., 2019; Ðanić et al., 2019; Đanić et al., 2021). The gut microbiome encodes enzymes which perform drug biotransformation, including reduction, hydrolysis, acetylation, deamination, dehydroxylation, decarboxylation, demethylation, deconjugation and proteolysis, making gut microbiota an important site of first-pass metabolism (Thiele et al., 2017). For instance, 5-aminosalicylic acid (5-ASA), the bioactive component of prodrug sulfasalazine used in IBD, is inactivated in the gut by bacterial arylamine N-acetyltransferases (Sandborn and Hanauer, 2003). The activity of these enzymes can differ up to tenfold between individuals, shedding light to the substantial interindividual differences in gut microbial metabolism that may influence the drug response (Deloménie et al., 2001). In addition, it has been shown that bile acids, which are direct products of the gut microbiota, may serve as reliable biomarkers for the prediction of outcomes for certain drugs (Kaddurah-Daouk et al., 2011; Lazarević et al., 2019; Lavelle and Sokol, 2020).

Drugs which undergo extensive enzymatic transformation by the host represent good candidates for the action of gut microbial enzymes and in some cases host and microbiota perform the same metabolic transformation (Zimmermann and Zimmermann-Kogadeeva, 2019). According to the Biopharmaceutics Drug Disposition Classification System (BDDCS) azathioprine is a drug of class 2, characterized by low solubility and extensive metabolism (Custodio et al., 2008). Following the oral administration of azathioprine, due to the low solubility and host factors, the absorption rate in the gastrointestinal tract is highly variable (Ding and Benet, 1979; Van Os et al., 1996; Gervasio et al., 2000). Accordingly, a large amount of the drug encounters with commensal bacteria in the small intestine, and especially in the large intestine, where the absorption of azathioprine was demonstrated to be significantly lower than in the upper gastrointestinal tract (Van Os et al., 1996). Since the pharmacokinetic characteristics of BDDCS Class 2 drugs can be clinically affected by metabolizing enzymes, whether the activity of gut microbial enzymes may affect the bioavailability of azathioprine *via* pre-uptake metabolism deserves investigation and further insight (Zhang et al., 2021). Furthermore, it has already been demonstrated that certain gut bacteria species are equipped with specific enzymes which could target the metabolic pathway of azathioprine (Cournoyer et al., 1998; Correia et al., 2016; Liu et al., 2017; Oancea et al., 2017; Crouwel et al., 2020). Hence, more in-depth insight into the microbial enzymes potentially responsible for the metabolism of azathioprine and thus leading to interindividual variations in dose-response profiles is needed to better understand azathioprine’s pharmacokinetics/pharmacodynamics characteristics and ultimately develop clinical guidelines for personalized pharmacotherapy. This study aimed at comprehensively reviewing gut bacteria which possess enzymes with the capability to metabolize azathioprine and proposing strategies for elucidating the contribution of these bacteria to the variability of drug responsiveness. As this field is lacking the overall systematic understanding of direct microbial enzymatic biotransformation of azathioprine, we intended, by a selection of gut microbiota-derived enzymes, to direct further exploration of metabolism of azathioprine and to highlight the role of gut microbiota in the drug response that will ultimately lead to the improvement of thiopurine therapy of IBD.

## Metabolic Pathway of Thiopurine Drugs as a Target for Gut Microbiota

Recently, the gut microbiota has often been referred to as the “invisible organ” due to its involvement in the modulation of the immune system, etiology of many diseases and metabolism of xenobiotics (Spanogiannopoulos et al., 2016). In the IBD, the microbial phyla compositions have been mainly examined in the context of the pathogenesis of the disease. The main differences in the gut microbiota between IBD patients and healthy individuals were demonstrated in the relative proportions of phyla within the Bacteroidetes, Firmicutes and Proteobacteria (Frank et al., 2007; Rehman et al., 2016). Less attention has been paid to the metabolic capacity of gut microbiota and its impact on the clinical effectiveness of drugs given in IBD (Ranjard et al., 2002; Becker et al., 2022).

**FIGURE 1:**
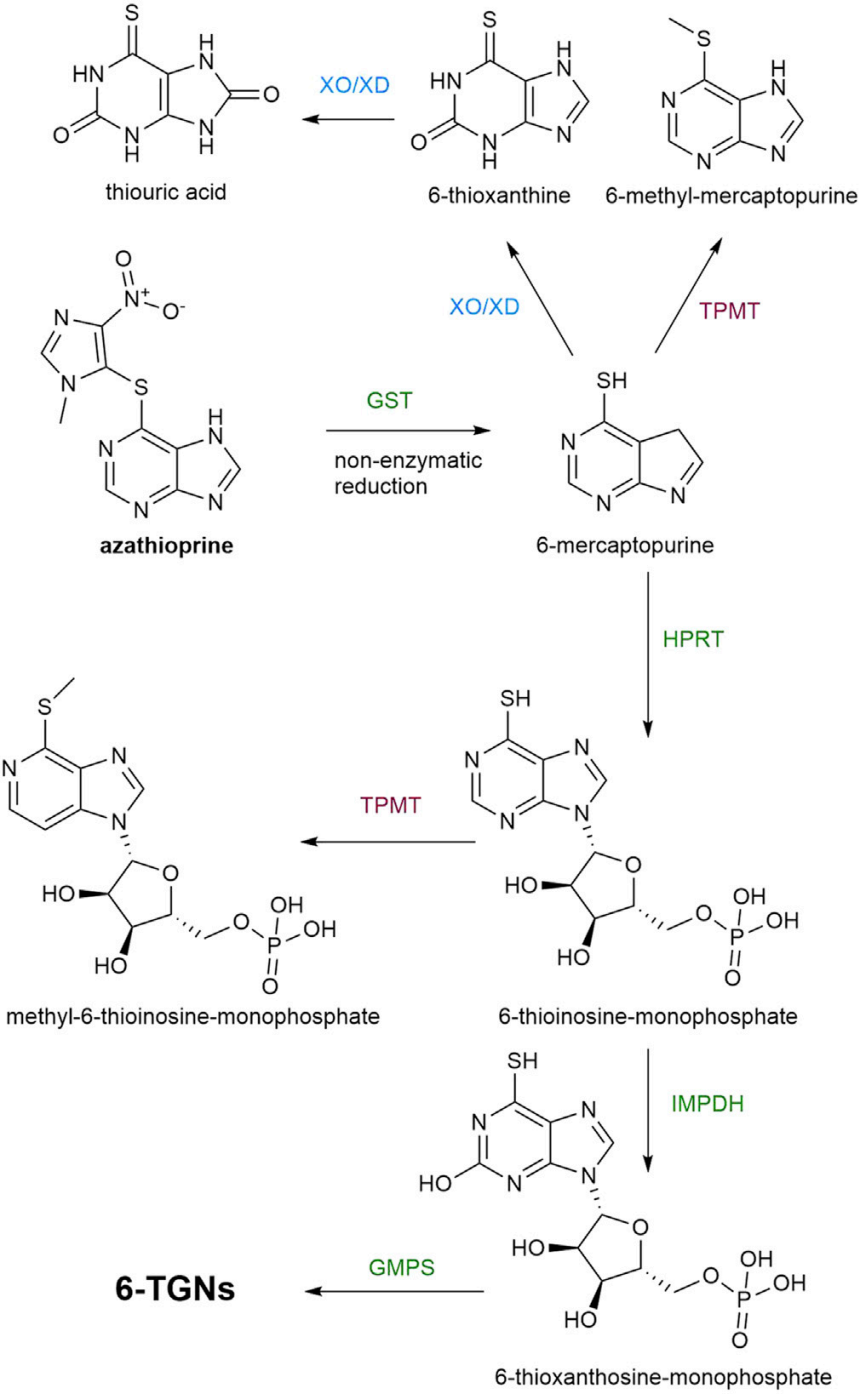
A metabolic pathway of azathioprine. Enzymatic reactions which lead to the synthesis of TGNs are marked with green color, while the pathways leading to the formation of inactive or toxic metabolites are marked with red and blue color. GST, glutathione S-transferases; TPMT, thiopurine S-methyltransferase; XO/XD, Xantine oxidase/xanthine dehydrogenase; HPRT, hypoxanthine phosphoribosyltransferase, IMPDH, inosine monophosphate dehydrogenase; GMPS, guanosine monophosphate synthase; TGNs, thioguanine nucleotides.

Thioguanine (TG) and the more conventional drugs, azathioprine and 6-MP, are immunomodulating agents used in IBD which have complex metabolism (Figure 1) and what is still to be elucidated is the involvement of gut microbiota in this process.

Azathioprine and 6-MP are prodrugs which require intracellular activation by hypoxanthine phosphoribosyltransferase (HPRT) into 6-thioinosine-monophosphate (TIMP) which is converted, by the action of inosinemonophosphate dehydrogenase (IMPDH), to 6-thioxanthosine-monophosphate and finally, by the action of guanosine monophosphate synthase (GMPS) into thioguanine nucleotides (TGNs), the active cytotoxic metabolites. Two other catabolic enzymatic pathways are known to compete with this anabolic metabolic pathway. Xantine oxidase (XO), proven to be present in both the intestinal mucosa and liver, converts 6-MP into 6-thioxanthine (6-TX) and subsequently into thiouric acid (TU). Another form of XO is xanthine dehydrogenase (XD). Both forms are interconvertible and catalyze the same reaction. Additionally, 6-MP is metabolized by TPMT into inactive 6-methyl-mercaptopurine (6-MMP). In comparison with 6-MP, azathioprine avoids first-pass metabolism in the gut more efficiently since azathioprine is not a substrate for XO, unlike 6-MP (Van Asseldonk et al., 2009). However, the bioavailability of azathioprine is 27–83% which is still highly variable (Van Os et al., 1996) and the important factor in limiting the systemic bioavailability of thiopurines that should not be neglected is the rich enzymatic machinery of gut microbiota. Formerly, the conversion of azathioprine to 6-MP and S-methyl-4-nitro-5-thioimidazole was thought to be a non-enzymatic reaction that occurs in the intestinal mucosa and liver. However, it has been demonstrated that 90% of this conversion is catalyzed by glutathione S-transferases (GSTs). For both pathways, the co-substrate used in this reaction is (reduced) glutathione (Van Asseldonk et al., 2009). The mechanism of action of human GSTs related to biotransformation of azathioprine includes a nucleophilic attack of the sulfur atom of deprotonated glutathione on the slightly electrophilic 5′ carbon atom in the imidazole moiety of azathioprine, forming 6-mercaptopurine and a glutathione-imidazole conjugate (Kurtovic et al., 2008b). Currently, human GST is considered as the most efficient enzyme in the bioactivation of prodrug azathioprine, thus the molecular docking studies of azathioprine-GST interactions have been performed with the aim of possible applications of targeted enzyme-prodrug therapy of diseases (Zhang W. et al., 2012; Modén and Mannervik, 2014). Moreover, the novel substrate for GST was synthesized which mimicked azathioprine in the reaction with glutathione in order to achieve efficient screening for variants of GSTs with higher catalytic activity toward azathioprine (Kurtovic et al., 2008a). A superfamily of bacterial GSTs is involved in a stunning variety of metabolic processes of the bacteria strains possessing these enzymes (Masip et al., 2006). Due to reported evidence of the bacterial GSTs interaction with certain xenobiotics, further studies on the ability of GSTs encoded in bacterial genomes to catalyze the conversion of azathioprine to 6-MP are required (Board et al., 2000; Vuilleumier and Pagni, 2002). GSTs are widely distributed in the phylum Proteobacteria, which is commonly increased in patients with IBD in respect to healthy controls (Vuilleumier and Pagni, 2002). Therefore, the contribution of members of this phylum present in the gut microbiota to the clinical responsiveness to azathioprine needs to be examined.

The other enzymes in the metabolic pathway of azathioprine which compete with the activation, TPMT and XO/XD, are clinically relevant enzymes (Ansari et al., 2008). Testing of the activity of these enzymes is frequently performed before the initiation of azathioprine therapy in order to tailor doses and prevent adverse effects (Lamb and Kennedy, 2019; Ding et al., 2021). Interestingly, a bacterial gene encoding a protein highly homologous to human TPMT was found and characterized in *Pseudomonas syringae* (Scheuermann et al., 2003), supporting reports about TPMT activity in *P. aeruginosa*, *fluorescens* and *ovalis* (Krynetski and Evans, 2003). Similarly, bacterial XO/XD homologous have gained considerable interest (Correia et al., 2016; Wang et al., 2016). According to the presence of XD, the leading phylum is Gammaproteobacteria which harbours the gut of IBD patients in a higher proportion (Wang et al., 2016). Liu et al. (Liu et al., 2017) have examined the effects of azathioprine, 6-MP and 5-ASA on the growth of fifteen different bacterial strains associated with IBD and performed in silico analysis of the presence of enzymes in these bacteria involved in the metabolism of thiopurine drugs. Interestingly, even though *Escherichia coli* possesses all enzymes required for conversion of azathioprine to TGNs, its growth was not significantly inhibited by azathioprine nor 6-MP. Conversely, the growth of *Campylobacter concisus* which does not possess enzymes GST and HPRT was significantly inhibited by azathioprine. These results indicated that inhibitory action of azathioprine and 6-MP on *C. concisus* does not occur through the conventional pathway.

Furthermore, data provided by Oancea et al. (Oancea et al., 2017) strongly supported the relevance of gut microbiota in thiopurine metabolism. Namely, they have demonstrated that specific bacteria belonging to the gut microbiota are able to convert 6-MP and TG, into therapeutically active TGNs, with threefold higher production in the case of TG. The reason for this difference in the amount of TGNs may be the less complex metabolic pathway of TG as the conversion of TG to TGNs is a direct result of HPRT enzyme activity. On the other hand, 6-MP conversion requires a few steps, as already mentioned (Figure 1), whereby the reaction catalyzed by the IMPDH could limit the production of TGN (Haglund et al., 2011). Nevertheless, these *in vitro* and *in vivo* studies conducted by Oancea et al. serve as a proof of how the gut microbiota can affect the pharmacokinetics of thiopurine drugs and may be the reason for interindividual variability in the therapeutic response (Atreya and Neurath, 2017). Bacterial species which are able to transform these drugs to TGNs are *Escherichia coli* (Proteobacteria), *Enterococcus faecalis* (Firmicutes) and *Bacteroides thetaiotaomicron* (Bacteroidetes) (Oancea et al., 2017). In comparison with azathioprine and 6-MP, TG may have a clinically faster onset of action but it is rarely used because of dose-related vascular liver toxicity which is a consequence of the rapid generation of TGNs in the portal circulation (Ward et al., 2017). Therefore, in this review we addressed the therapeutic efficacy of azathioprine, which is more frequently used than TG in clinical practice, from the aspect of its complex metabolic pathway as a potential site for the powerful enzymatic activity of bacteria belonging to gut microbiota.

## Selection of Gut Bacterial Species—Potential Candidates Responsible for the Biotransformation of Azathioprine

The chemical structure of certain drugs, in particular their functional groups (such as lactones, nitro, azo, sulfhydryl and urea groups), predispose these compounds for metabolic modifications by gut bacteria. As Zimmermann et al. (Zimmermann and Zimmermann-Kogadeeva, 2019) have pointed out, the drugs metabolized by most of the bacteria (except Proteobacteria) contain a nitro or azo group, which is prone to the reduction in anaerobic metabolism, while drugs which are specifically metabolized by Bacteroidetes contain ester or amide groups that can be hydrolyzed. Such chemical modifications of drugs by gut microbiota can lead either to their activation, inactivation or even to the toxification (Stojančević et al., 2014). In UC therapy, a most typical example of the drug biotransformed by gut microbial enzymes is sulfasalazine. The azo bond of prodrug sulfasalazine is attacked by gut bacterial azoreductases, releasing 5-ASA and sulfapyridine. 5-ASA is an active compound while the latter one causes side effects such as nausea, anorexia and skin rash (Franzin and Stefančič, 2021).

As it was demonstrated in the studies mentioned in the previous section, specific gut bacterial species could carry a complete metabolic pathway of 6-MP independently of the host enzymes. Nevertheless, converting azathioprine to 6-MP requires GST enzymatic activity which certainly expands the library of potential gut bacterial candidates for the metabolism of azathioprine. Also, the recent study showing how a drug metabolite produced by one bacteria species could be a substrate for the biotransformation by another bacteria species, causing the variable interpatient response to the drug, indicates how important is to identify the specific species and pathways that metabolize the drug (Rekdal et al., 2019).

The enzymes activating and inactivating production of TGNs in the metabolic pathway of azathioprine which should be considered as the potential target for the gut microbial enzymes are: GST, TPMT, XO/XD, HPRT, IMPDH, GMPS.

In order to identify bacteria that encode candidate enzymes required for the metabolism of azathioprine we performed the research by reviewing available literature and by using publicly available enzyme databases as BRENDA, MetaCyc, National Center for Biotechnology Information (NCBI) protein database and Universal Protein Resource (UniProt). In Table 1 we have reviewed the bacteria by the criteria of possession of enzymes which could catalyze the transformation of azathioprine in the gut and contribute to the treatment outcome. The bacteria species addressed in Table 1 are the members of commensal gut microbiota or pathobionts involved in IBD.

**TABLE 1:** Classification of gut bacteria which possess enzymes involved in the metabolic pathway of azathioprine.

Phylum	Class	Order	Family	Genus	Species	Enzyme	Reference
Proteobacteria	Gammaproteobacteria	Enterobacterales	Enterobacteriaceae	*Escherichia*	*Escherichia coli*	GST	Vuilleumier and Pagni, (2002); Wang et al. (2009)
						XO/XD	Xi et al. (2000); Leimkühler, (2014); Liu et al. (2017)
						HPRT	Eng et al. (2016)
						IMPDH	Gilbert et al. (1979); Pimkin and Markham, (2009)
						GMPS	Tesmer et al. (1994)
				*Enterobacter*	*Enterobacter cloacae*	GST	Piccolomini et al. (1989)
						XO	Machida and Nakanishi, (1981)
		Pseudomonadales	Pseudomonadaceae	*Pseudomonas*	*Pseudomonas fluorescens*	TPMT	Krynetski and Evans, (2003)
						GST	Zablotowicz et al. (1995)
	Epsilonproteobacteria	Campylobacterales	Campylobacteraceae	*Campylobacter*	*Campylobacter concisus*	IMPDH	Liu et al. (2017)
						GMPS	Liu et al. (2017)
Firmicutes	Bacilli	Lactobacillales	Enterococcacae	*Enterococcus*	*Enterococcus faecalis*	XD	Srivastava et al. (2011)
						HPRT	Gaca et al. (2013); Liu et al. (2017)
						IMPDH	Riboulet-Bisson et al. (2008)
						GMPS	Liu et al. (2017)
		Bacillales	Bacillaceae	*Bacillus*	*Bacillus subtilis*	HPRT	Christiansen et al. (1997)
Bacteroidetes	Bacteroidia	Bacteroidales	Bacteroidaceae	*Bacteroides*	*Bacteroides thetaiotaomicron*	HPRT	Franzin and Stefančič, (2021)
						IMPDH	Franzin and Stefančič, (2021)
						GMPS	Pimkin and Markham, (2009); Franzin and Stefančič, (2021)
					*Bacteroides fragilis*	HPRT	Zhang et al. (2012a); Franzin and Stefančič, (2021)
						IMPDH	Liu et al. (2017); Franzin and Stefančič, (2021)
						GMPS	Liu et al. (2017); Franzin and Stefančič, (2021)
				*Phocaeicola*	*Bacteroides vulgatus*	GST	Liu et al. (2017)
						XO	Liu et al. (2017)
						HPRT	Liu et al. (2017)
						IMPDH	Liu et al. (2017)
						GMPS	Liu et al. (2017)

As shown in Table 1, potential candidates for microbial structural modifications of azathioprine belong to the phyla whose compositions were demonstrated to be disturbed in patients with IBD (Proteobacteria, Firmicutes, Bacteroidetes). An exception, not considered as a typical gut-associated species, is *P. fluorescens* which was shown to possess bacterial GST and TPMT*. P. fluorescens* and other members of the family Pseudomonadaceae are rare components of fecal bacteria of the colon. However, PCR assays for the *P. fluorescens*-specific I2 sequence are usually positive for mucosa of the ileum (both healthy and IBD individuals) and colon (CD) suggesting that *P. fluorescens* may be the low-level commensal of ileal mucosa in IBD and may expand its colonization to susceptible colonic mucosa in CD (Wei et al., 2002). Therefore, this species should be also considered in the analysis of the enzymatic transformation of azathioprine by gut microbiota. Similarly, *Bacillus subtilis*, which is usually considered a soil organism, has been isolated from the human gut and thought to be adapted to life within the human gastrointestinal tract (Hong et al., 2009). Furthermore, human gastrointestinal isolates of *B. subtilis* have shown increased sporulation compared to traditional lab-grown isolates of *B. subtilis* indicating that this strain in the human gut had been previously under-represented (Tam et al., 2006; Egan et al., 2021).

Although the bacteria species selected in Table 1 appeared to encode genes for the enzymes required for the metabolism of azathioprine, it remains to confirm their enzymatic activity.

## Microbiota-Drugs Interactions: Future Perspective of Personalized Thiopurine Therapy in IBD

Since there is no cure for IBD and the challenging process of designing and introducing new drugs into the market, it is absolutely worthwhile to use the maximum potential of conventional therapy. Thus, predictors of responsiveness to the thiopurine therapy and adverse effects need to be elucidated. Lately, the investigation of factors that cause interpersonal variability has shifted the focus from the human genome analysis to the role of gut microbiota in drug response and toxicity. Various models have been applied to study gut microbiota-drug interactions (Liu et al., 2017; Oancea et al., 2017; Xu et al., 2018; Dhurjad et al., 2022). In a recently published study, Zimmermann et al. (Zimmermann et al., 2019) showed that even two-thirds of 271 studied drugs have been metabolized by at least one strain of human gut bacteria, confirming that the link between the gene content and metabolic activity of gut bacteria directly reflects on interindividual differences in therapeutic outcome. Accumulating evidence from different *in vivo*, *in vitro* and in silico studies mentioned in this review has provided a strong rationale for thoroughly designed research of the microbiota-driven metabolism of azathioprine which could lead to firm conclusions regarding the drug outcome (Ogita et al., 2015; Liu et al., 2017; Oancea et al., 2017). The novel experimental setup for mapping the ability of the human gut microbiome to metabolize small molecule drugs showed that azathioprine after incubation with microbiota was no longer detectable, classifying it to the group of drugs prone to microbiome-derived metabolism (Javdan et al., 2020). What makes this approach unique is the use of subject-personalized microbial communities rather than the use of monocultures of a selected set of representative species. This is of vital importance because the expression of genes for microbial enzymes differs sustainably between a strain grown in monoculture versus heterogeneous bacterial communities in which strains interact in complex patterns (Javdan et al., 2020; Lucafò and Franzin, 2020). Nevertheless, in order to generate high-quality predictive models for thiopurine therapy of IBD, this type of workflow which has merged computational and *in vitro* techniques must go along with well-designed human *in vivo* studies. Next-generation sequencing is a well-established technology for identifying human gut microbial fingerprints from fecal samples (Malla et al., 2018; Sanchis-Artero and Martínez-Blanch, 2020). Microbiota perturbations in IBD patients and various environmental factors that can influence the diversity of microbiota indicate that the investigation of microbial metabolism of azathioprine should be performed in precisely chosen study populations. The experimental design should also include a questionnaire concerning diet, lifestyle, use of probiotics, antibiotics and other factors affecting gut microbiota. Moreover, azathioprine itself may affect the composition of gut microbiota, thus, it would be thought-provoking to analyze longitudinal microbiota changes in IBD patients before and throughout the use of azathioprine. Related to this, the development of microfluidic gut-on-a-chip has opened a new avenue for studying microbial enzymatic pathways and the drug impact on microbiota on *in vitro* IBD-specific models (Lee et al., 2016; Guo et al., 2018). In particular, employing human samples from different cohort groups of IBD patients on this device which mimics an *in vivo*-like intestinal microenvironment could facilitate translational research.

A growing body of evidence on gut microbiota-drug interactions suggests that the gut microbial signature is a powerful tool for the prediction of therapeutic outcomes and represents the future of precision medicine in IBD. Therefore, novel models for studying this field are urgently needed. New approaches would ultimately pave the way for personalized pharmacotherapy based on the patient’s microbiota and the associated interactions that may lead to modifications in dosage or therapy.

Due to the lack of systematic information regarding azathioprine metabolism by gut microbiota, herein we accumulated consistent findings of microbial enzymes which could potentially metabolize azathioprine and impact the clinical outcome. This review provides a solid basis for future studies on the effects of gut microbiota on azathioprine, by eliciting detailed pathways of microbiota-drug interactions of which an understanding fosters the required rationale for personalized pharmacotherapy to eventuate into clinical practice guidelines.
